# Data-driven quantum approximate optimization algorithm for power systems

**DOI:** 10.1038/s44172-023-00061-8

**Published:** 2023-03-09

**Authors:** Hang Jing, Ye Wang, Yan Li

**Affiliations:** 1 Department of Electrical Engineering, The Pennsylvania State University, University Park, PA 16802 USA; 2 Duke Quantum Center, Duke University, Durham, NC 27708 USA; 3 Department of Electrical & Computer Engineering, Duke University, Durham, NC 27708 USA

**Keywords:** Electrical and electronic engineering, Quantum information

## Abstract

Quantum technology provides a ground-breaking methodology to tackle challenging computational issues in power systems. It is especially promising for Distributed Energy Resources (DERs) dominant systems that have been widely developed to promote energy sustainability. In those systems, knowing the maximum sections of power and data delivery is essential for monitoring, operation, and control. However, high computational effort is required. By leveraging quantum resources, Quantum Approximate Optimization Algorithm (QAOA) provides a means to search for these sections efficiently. However, QAOA performance relies heavily on critical parameters, especially for weighted graphs. Here we present a data-driven QAOA, which transfers quasi-optimal parameters between weighted graphs based on the normalized graph density. We verify the strategy with 39,774 expectation value calculations. Without parameter optimization, our data-driven QAOA is comparable with the Goemans-Williamson algorithm. This work advances QAOA and pilots its practical application to power systems in noisy intermediate-scale quantum devices.

## Abstract (web summary)

Data-Driven Quantum Approximate Optimization Algorithm for Power Systems Quantum Approximate Optimization Algorithms can enhance the monitoring, operation, and control of Distributed Energy Resources. Li and coworkers reduce the computational effort required for training these algorithms by efficiently obtaining algorithm parameters.

## Introduction

Quantum technology is emerging as a new hope to address challenging computational tasks in power systems, including quantum chemistry simulation for new type batteries^1–3^, efficient power system analysis by solving linear systems of equations^4–8^, forecasting highly chaotic systems^9^, scheduling and dispatching power grids^10^, unit commitment^11^, optimal reconfiguration of distribution grids^12^, etc. However, the existing algorithms require substantial quantum resources, limiting their near-term utilization on noisy intermediate-scale quantum (NISQ) devices^13^. Even though specific instances of quantum algorithms have been demonstrated on various quantum processors with tens of qubits^14–16^, practical applications to address power system problems will still require further advances in algorithmic design.

**Fig. 1:**
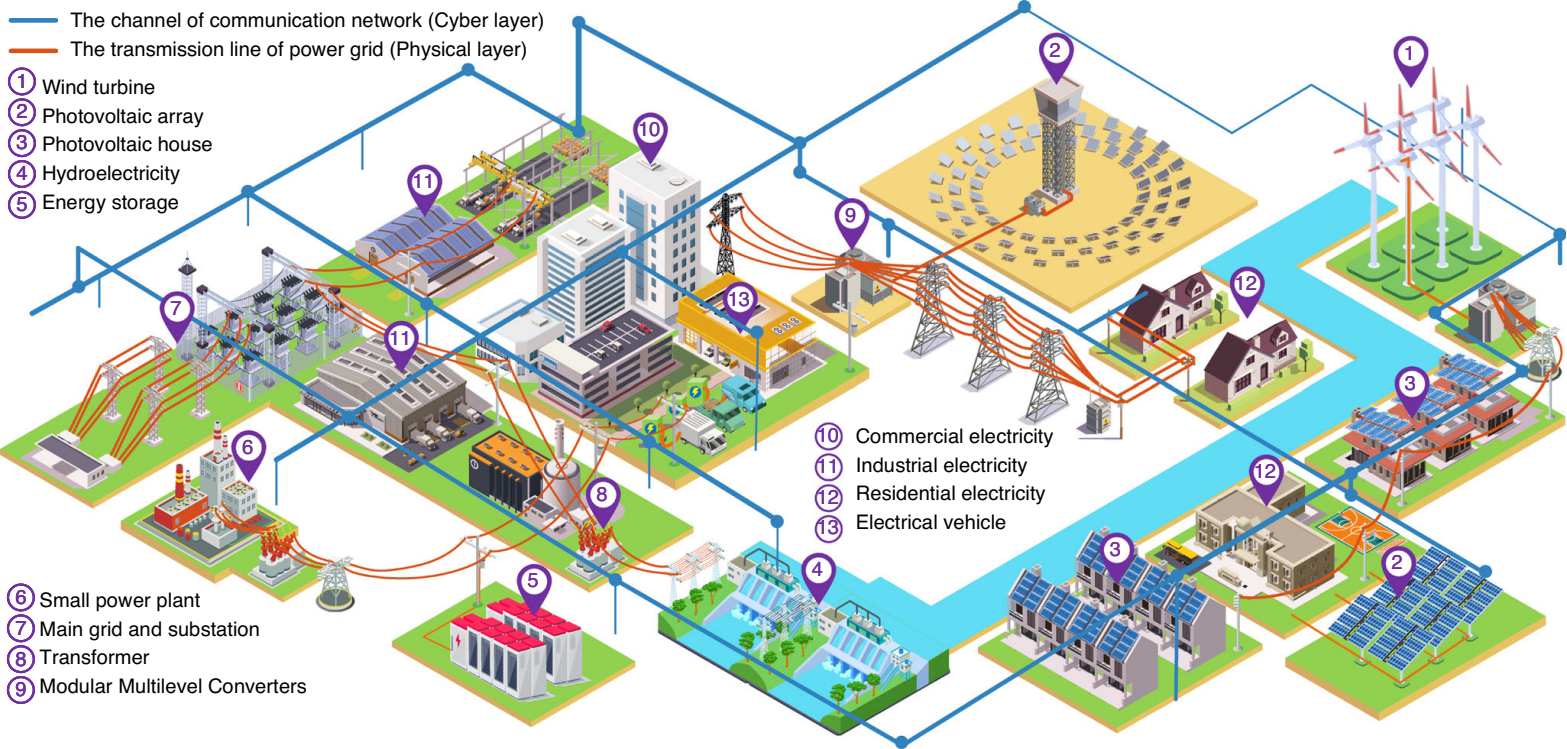
Illustration of the cyber-physical power system. It includes physical layer and cyber layer. The physical layer is Distributed Energy Resources (DERs) dominant power grid. The cyber layer is used for the communication among DERs and the control center for the system’s operation and control.

In power systems, one emerging quantum application is to analyze the Distributed Energy Resources (DERs) dominant power system, which provides a potent solution to seek an edge toward energy sustainability. In Fig. 1, we illustrate a typical DER dominant cyber-physical power system includes physical layer and cyber layer. The physical layer is energized by DERs and the cyber-layer enables the communication among DERs^17^ through the advanced metering infrastructure and Internet of Things system^18^ for system coordination and control^19^.

To improve the resiliency of the system, it is critical to efficiently obtain the maximum sections of power energy in the physical layer and data traffic in the cyber layer^20–22^. Mathematically, finding the maximum section of power energy or data traffic is to solve the Max-Cut problem, which is an NP-hard issue^23^. Therefore people implement classical approximation algorithms^24–27^ to address the Max-Cut problem in practical applications. However, for specific instances, classical algorithms can only guarantee an approximation ratio of 0.878^24,28^.

The Quantum Approximate Optimization Algorithm (QAOA), a hybrid quantum-classical algorithm, is expected to obtain better approximate solutions than any existing classical algorithms^29,30^. QAOA utilizes a classical computer trains the parameters for quantum circuit^29^. The parameterized quantum circuit approximates the adiabatic evolution from an initial Hamiltonian, whose ground energy state is easy to prepare, to a final Hamiltonian, whose ground energy state encodes the solution of the Max-Cut problem. With an ideal approximation, people expect to obtain the exact solution of the Max-Cut problem with high probabilities^31^. Consequently, the parameters involved in the quantum circuit play an essential role in getting high-quality approximations^32–34^. However, how to efficiently obtain appropriate parameters is still an open question.

This work presents a data-driven QAOA with parameter transfer strategy to tackle the challenging issue of efficiently obtaining appropriate parameters for QAOA. Therefore, the data-driven QAOA enables efficient search of the maximum sections of power delivery and data traffic in cyber-physical power systems. The contributions of this work are summarized below. First, a parameter transfer strategy based on the normalized graph density is developed for QAOA on generic weighted graphs. Through the transfer strategy, quasi-optimal parameters can be obtained for the target (new) graphs from the seed (existing) graphs. Those parameters can then be either directly applied or used as an initial guess for further optimization. The transfer strategy is designed to be extendable, allowing new verified graph-parameter pairs to be added to the transfer database. Therefore, we can enable efficient QAOA computation. Second, based on the transfer strategy, the data-driven QAOA framework is established. We have numerically justified the effectiveness of the strategy through evaluating the QAOA’s performance on 1710 random instances with the transferred parameters and the ones after optimization. Third, we also perform the data-driven QAOA in practical power systems to get their maximum sections. Simulations on 996 case studies have validated that the data-driven QAOA can efficiently obtain comparable results to the Goemans-Williamson (GW) algorithm. It sheds light on the online computation and analysis. Additionally, the study on the near-term achievable noise of quantum processor shows that it is negligible to our data-driven QAOA method. Overall, this work reduces the computational effort required for training QAOA parameters, advances the development of QAOA, and highly promotes its wide applications for solving engineering problems. As a practical quantum application, it is feasible shortly in the NISQ era to address problems in power systems. Recently, we became aware of a similar work by Shaydulin et al. about the transferability between weighted graphs^35^, which was carried out independently.

## Results

### Maximum sections problem formulation

In the power system, the maximum power (data) section is defined as an edge collection ${{{{{{\mathcal{C}}}}}}}^{* }$ of the graph *G* = (*V*, *E*), which is modeled from the physical (cyber) layer. The collection ${{{{{{\mathcal{C}}}}}}}^{* }$ has the following three properties: First, ${{{{{{\mathcal{C}}}}}}}^{* }$ is a subset of the edge set *E*, namely, ${{{{{{\mathcal{C}}}}}}}^{* }\subseteq E$. Second, the reduced graph $\bar{{{\mathrm{G}}}}=(V,{{{{{{\mathcal{C}}}}}}}^{* })$ is a bipartite graph. Third, as shown in (1), the summation of the edge weights in ${{{{{{\mathcal{C}}}}}}}^{* }$ is the supremum of the edge summation of all possible collections ${{{{{\mathcal{C}}}}}}$, which satisfy the first two properties.

1 $$\mathop{\sum}\limits_{\left\langle i,j\right\rangle \in {{{{{{\mathcal{C}}}}}}}^{* }}{w}_{ij}=\mathop{\sup }\limits_{{{{{{\mathcal{C}}}}}}\subseteq E}\mathop{\sum}\limits_{\left\langle i,j\right\rangle \in {{{{{\mathcal{C}}}}}}}{w}_{ij}$$

It is critical to efficiently obtain the maximum sections because of three reasons. First, the maximum power section offers a cost-effective way to monitor the dynamics and power delivery capability of the physical system, especially when the system’s operation frequently changes caused by intentional/unintentional disturbances, such as the fluctuations of DERs and the changes of system topology due to the join or removal of subsystems (e.g., microgrids)^20^. Second, the maximum power sections cast light on the dynamic system’s control and operations. Dispatchable DERs can be coordinated for controlling the electric power over the maximum section to improve the whole system’s operation^21^. Third, the maximum data traffic sections provide an insight into enhancing the overall power system’s resilience through strategically designing and managing the communication network^22,36^, e.g., packet routing and traffic control.

Mathematically, finding the maximum section is to solve a Max-Cut problem of a weighted graph *G* = (*V*, *E*), where ∣*V*∣ = *n* is the vertex number, ∣*E*∣ = *m* is the edge number, and *w*_*ij*_ represents the normalized weight of the edge $\langle i,j\rangle \in E$, where ${{{{{\rm{Max}}}}}}({w}_{ij})=1$. The Max-Cut solutions are identical before and after normalization. The edge weight is obtained via power flow calculation for the physical layer and means the data traffic in the cyber layer. Modeling details are provided in the Methods section “Modeling the Power System”.

The objective is to find a subset *S* ⊂ *V* that maximizes $\mathop{\sum }\nolimits_{i\in S,j\notin S}^{}{w}_{ij}$ for cyber or physical layers, respectively. Suppose an *n*-bit string *Z* = *z*_1_ ⋯ *z*_*i*_ ⋯ *z*_*j*_ ⋯ *z*_*n*_ ∈ {−1, 1}^*n*^ can denote the status of vertices *V*, showing each bit *z*_*i*_ will be equal to 1 if the *i*^th^ vertex is in the subset *S*, otherwise be − 1. We can exhibit the partition of vertices for obtaining the maximum section. Thus, the classical cost function of the Max-Cut problem can be defined as,

2 $$C(Z)=\mathop{\sum}\limits_{\left\langle i,j\right\rangle \in E}{w}_{ij}\frac{1-{z}_{i}{z}_{j}}{2}=\mathop{\sum}\limits_{\left\langle i,j\right\rangle \in E}{w}_{ij}{C}_{ij}(Z),$$

where *C*_*ij*_(*Z*) represents the contribution of *w*_*ij*_ to the cost function. The Max-Cut problem translates into finding the *n*-bit string *Z* to maximize the cost function *C*(*Z*). Given the *n*-bit string *Z*, we define the approximation ratio to be *C*(*Z*)/*C*(*Z*_Max-Cut_), where *Z*_Max-Cut_ is the exact Max-Cut solution. The goal of approximate algorithms is to find the solution with a high approximation-ratio.

### QAOA for max-cut problem

On quantum computers, we use *n* quantum bits (qubits) $\left\vert Z\right\rangle =\vert {z}_{1}\cdots {z}_{i}\cdots {z}_{j}\cdots {z}_{n}\rangle$ to represent the status of *n* vertexes. Each qubit $\vert {z}_{i}\rangle$ can be a superposition of quantum states $\left\vert 0\right\rangle$ and $\left\vert 1\right\rangle$, denoted as $\vert {z}_{i}\rangle ={a}_{i}\vert 0\rangle +{b}_{i}\vert 1\rangle$, where $\left\vert 0\right\rangle$ and $\left\vert 1\right\rangle$ are the eigenstates of the Pauli-Z operator *σ*^*z*^ with the eigenvalues of 1 and − 1 respectively. When we measure the qubit in the computational basis, which is *z* basis, according to the quantum mechanics, the qubit could collapse to the state $\left\vert 0\right\rangle$ with probability of ${\vert {a}_{i}\vert }^{2}$ and the state $\left\vert 1\right\rangle$ with probability of ${\vert {b}_{i}\vert }^{2}$. Therefore, unlike classical computers, the measurement results could vary even though the qubit is identical at each execution. If we consider that measuring $\left\vert 0\right\rangle$ represents *z*_*i*_ = 1 and measuring $\left\vert 1\right\rangle$ represents *z*_*i*_ = − 1, we can obtain various *n*-bit strings *Z* = *z*_1_ ⋯ *z*_*i*_ ⋯ *z*_*j*_ ⋯ *z*_*n*_ ∈ {−1, 1}^*n*^ and calculate *C*(*Z*) in (2) after every single quantum computer execution.

On the other hand, we could obtain the deterministic *n*-bit string *Z*_*k*_ out of the measurements on 2^*n*^*n*-qubit eigenstates in the computational basis, denoted as $\vert {Z}_{k}\rangle$ with $\vert {z}_{k,i}\rangle =\left\vert 0\right\rangle$ or $\left\vert 1\right\rangle$ and ${z}_{k,i}=\langle {z}_{k,i}\vert {\sigma }_{i}^{z}\vert {z}_{k,i}\rangle$. Therefore, we can have

3 $$C({Z}_{k}) 	 = \mathop{\sum}\limits_{\left\langle i,j\right\rangle \in E}{w}_{ij}\frac{1-{z}_{k,i}{z}_{k,j}}{2}\\ 	 =\mathop{\sum}\limits_{\left\langle i,j\right\rangle \in E}{w}_{ij}\frac{1-\langle {z}_{k,i}\vert {\sigma }_{i}^{z}\vert {z}_{k,i}\rangle \langle {z}_{k,j}\vert {\sigma }_{i}^{z}\vert {z}_{k,j}\rangle }{2}\\ 	 =\left\langle {Z}_{k}\right\vert {H}_{{{{{{\rm{C}}}}}}}\left\vert {Z}_{k}\right\rangle \\ 	 \equiv C\left(\left\vert {Z}_{k}\right\rangle \right),$$

where

4 $${H}_{{{{{{\rm{C}}}}}}}=\mathop{\sum}\limits_{\left\langle i,j\right\rangle \in E}{w}_{ij}\frac{I-{\sigma }_{i}^{z}{\sigma }_{j}^{z}}{2}.$$

We consider $C(\left\vert Z\right\rangle )=\left\langle Z\right\vert {H}_{{{{{{\rm{C}}}}}}}\left\vert Z\right\rangle$ as the quantum analog of *C*(*Z*). Then 2^*n*^ classical cost functions *C*(*Z*_*k*_) are one-on-one mapped to 2^*n*^ quantum cost functions $C(\vert {Z}_{k}\rangle )$. The Max-Cut problem translates into finding the quantum state $\vert {Z}_{k}\rangle$ to maximize the cost function $C(\vert {Z}_{k}\rangle )$.

The 2^*n*^$\vert {Z}_{k}\rangle$ states form the complete basis of the 2^*n*^ Hilbert space for *n*-bit quantum states. Therefore we can decompose an arbitrary state $\left\vert Z\right\rangle$ into a linear combination of $\vert {Z}_{k}\rangle$, denoted as $\left\vert Z\right\rangle =\mathop{\sum }\nolimits_{k = 1}^{{2}^{n}}{\alpha }_{k}\left\vert {Z}_{k}\right\rangle$ with $\mathop{\sum }\nolimits_{k = 1}^{{2}^{n}}{\left\vert {\alpha }_{k}\right\vert }^{2}=1$. The quantum cost function of an arbitrary state $\left\vert Z\right\rangle$ can be written as

5 $$C(\left\vert Z\right\rangle ) 	 =\left\langle Z\right\vert {H}_{{{{{{\rm{C}}}}}}}\left\vert Z\right\rangle \\ 	 =\left(\mathop{\sum }\limits_{k=1}^{{2}^{n}}{\alpha }_{k}^{* }\left\langle {Z}_{k}\right\vert \right){H}_{{{{{{\rm{C}}}}}}}\left(\mathop{\sum }\limits_{k=1}^{{2}^{n}}{\alpha }_{k}\left\vert {Z}_{k}\right\rangle \right)\\ 	 =\mathop{\sum }\limits_{k=1}^{{2}^{n}}{\left\vert {\alpha }_{k}\right\vert }^{2}C\left(\left\vert {Z}_{k}\right\rangle \right).$$

Since $C(\left\vert Z\right\rangle )=\left\langle Z\right\vert {H}_{{{{{{\rm{C}}}}}}}\left\vert Z\right\rangle \ge 0$, we have $\max C(\left\vert Z\right\rangle )=\underset{k=1}{\overset{{2}^{n}}{\max }}C(\vert {Z}_{k}\rangle )$. Notably, in quantum mechanics, $C(\left\vert Z\right\rangle )=\left\langle Z\right\vert {H}_{{{{{{\rm{C}}}}}}}\left\vert Z\right\rangle$ is the expectation value of system energy for a quantum system described by Hamiltonian *H*_C_. The Max-Cut problem translates into finding the maximum energy state for the quantum system described by Hamiltonian *H*_C_.

QAOA utilizes a quantum circuit running on the quantum computer to approximate an adiabatic evolution from the maximum energy state of an initial Hamiltonian, *H*_B_, to the maximum energy state of the final Hamiltonian, *H*_C_. For the Max-Cut problem, we particular define the *H*_B_ as

6 $${H}_{{{{{{\rm{B}}}}}}}=\mathop{\sum }\limits_{j=1}^{n}{\sigma }_{j}^{x}.$$

According to adiabatic theorem^37^, with an ideal approximation, we expect to obtain the maximum energy state of *H*_C_, which leads to the exact Max-Cut solution, with a high probability.

To implement QAOA on quantum computers, we first prepare the maximum energy state of *H*_B_, ${\left\vert +\right\rangle }^{\otimes n}$, as the initial state for the quantum circuit. Then we run the quantum circuit with 2*p* trainable parameters $\gamma =({\gamma }_{1},{\gamma }_{2},\ldots ,{\gamma }_{p})$ and $\beta =({\beta }_{1},{\beta }_{2},\ldots ,{\beta }_{p})$ to approximate the *p*-step Trotter expansion of the adiabatic evolution. We measure the output state, obtain the classical *n*-bit string, and estimate the quantum cost function using (5) with multiple executions. After that, we use the classical-quantum hybrid optimizer iterating 2*p* parameters to maximize the quantum cost function. Ideally, when *p* tends to infinity, the probability of obtaining the exact Max-Cut solution will tend to be 1. Even with a finite *p*, measuring the final state $\left\vert Z\right\rangle$ of the optimized circuit could generate high approximation-ratio solutions. More details are discussed in the Methods section “Adiabatic Approximation with QAOA”.

### Data-driven QAOA

People have studied QAOA’s efficiency and accuracy in regular graphs with constant circuit depth^29,38–41^. On the other side, the performance of QAOA on generic weighted graphs is an open question and challenging to estimate rigorously^42^. Heuristic strategies show potentials to find quasi-optimal parameters with high approximation-ratio solutions, a claim backed by numerical evidences^32,38,40^. However, researchers have not exhaustively explore the heuristic strategies on generic weighted graphs^42^.

Our data-driven QAOA for generic weighted graphs can provide a high approximation-ratio solution without parameter optimization to avoid expensive computational effort. The data-driven QAOA is based on normalized weighted graph density *D*^43^, which is defined as,

7 $$D=\mathop{\sum}\limits_{\left\langle i,j\right\rangle \in E}\frac{2{w}_{ij}}{n(n-1)}.$$

**Fig. 2:**
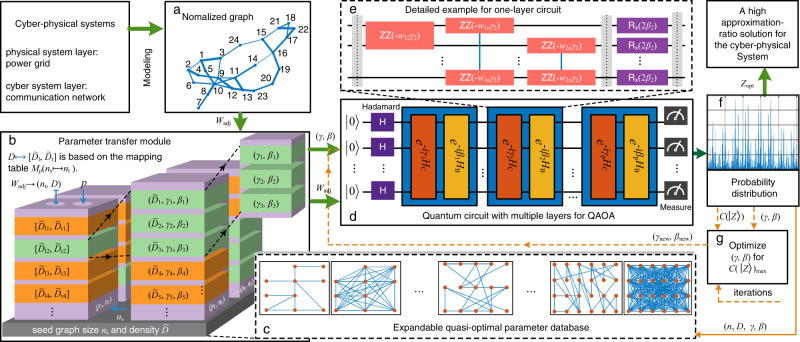
Schematic of the proposed data-driven Quantum Approximate Optimization Algorithm (QAOA) for obtaining the maximum sections of the cyber-physical systems. **a** Modeling the cyber-physical system into normalized weighted graphs. **b** The parameter transfer module that obtains the quasi-optimal parameters (*γ*, *β*). **c** The expandable quasi-optimal parameter database that stores and updates the mapping tables for (**b**). **d** The quantum circuit with multiple layers by using the transferred parameters (*γ*, *β*). **e** The detailed example for one-layer circuit in (**d**). **f** The probability distribution by measurement for computing the cost function and selecting a high approximation-ratio solution. **g** Optimize the parameters for better performance and extend the database in (**c**) if needed.

The data-driven QAOA includes five steps as shown in Fig. 2 and introduced as follows, where an innovative parameter transfer strategy is the key idea.

Step 1: Formulate the search of maximum section to the Max-Cut problem of the normalized weighted graph and calculate its *D*.

Step 2: Obtain the quasi-optimal parameters (*γ*, *β*) based on *D* via the parameter transfer strategy, and then pass these parameters to the quantum processors. The transfer strategy works for generic weighted graphs.

Step 3: Construct the quantum circuit with the adjacency matrix *W*_adj_ and parameters (*γ*, *β*), and run it in a quantum processor. Then, measure the output state of the quantum circuit to get the probability distribution and calculate the cost function value.

Step 4: Optimize the parameters by the classical optimizer for a better result when necessary. Step 4 is optional.

Step 5: Expand the database by adding more pairs denoted by (*n*, *D*, *γ*, *β*) from verified cases, to provide more quasi-optimal parameters. Step 5 is optional.

Decent initial guesses obtained in Step 2 can also help to handle noise-free barren plateaus, which are linked to random parameter initialization^44^. These initial guesses also reduce the iterations between the classical optimizer and the quantum processor, saving the running time of the algorithm. For the proposed data-driven QAOA, we also provide the pseudocode Algorithm 1 in “Supplementary Methods”.

### Parameter transfer strategy

The essential idea of the data-driven QAOA in Fig. 2 is the parameter transfer strategy, as summarized in the Step 2. It includes the following three substeps, which highly improves the effectiveness of transfer.

Substep 1: Establish the initial database. Several seed graphs are randomly generated, with their normalized graph densities spreading over [0, 1]. Considering the small size of these graphs, we can calculate the quasi-optimal parameters (*γ*, *β*)^40,41^ as shown in Methods section “Optimization of Parameters”. These parameters provide potentially quasi-optimal parameters for new graphs. This feature is particularly appealing to relatively larger target graphs. The database can then be established, based on the pairs (*n*, *D*, *γ*, *β*).

Substep 2: Develop the mapping table. The mapping table is designed for transferring quasi-optimal parameters from seed graphs to target graphs with the same circuit layer number *p*. For creating the mapping table, several target graphs are also randomly generated, with normalized graph densities spread over [0, 1]. With the parameters obtained from the seed graphs, QAOA calculation is performed for each target graph to get the cost function value $C(\left\vert Z\right\rangle )$. Assume there are ${{{{{\mathcal{N}}}}}}$ seed graphs with *n*_s_ vertices and ${{{{{\mathcal{M}}}}}}$ target graphs with *n*_t_ vertices, the values of $C(\left\vert Z\right\rangle )$ are then organized into a ${{{{{\mathcal{N}}}}}}\times {{{{{\mathcal{M}}}}}}$ matrix *M*_*p*_(*n*_s_ ↦ *n*_t_), i.e., the mapping table. In the table, each column is corresponding to one target graph and each row is corresponding to one seed graph. Note a mapping table only needs to be prepared once in advance for the same *p*, *n*_s_, *n*_t_.

Substep 3: Transfer parameters to new graphs. For a new graph with normalized graph density ${D}^{{\prime} }$ and size ${n}_{{{{{{\rm{t}}}}}}}^{{\prime} }$, several appropriate seed graphs will be selected from the mapping table ${M}_{p}({n}_{{{{{{\rm{s}}}}}}}\mapsto {n}_{{{{{{\rm{t}}}}}}}^{{\prime} })$, whose size *n*_s_ needs to be equal or close to ${n}_{{{{{{\rm{t}}}}}}}^{{\prime} }$. Then, in the mapping table, one (or more) column, whose corresponding *D* is equal or close to the new graph’s ${D}^{{\prime} }$, will be selected. Since each entry of this column is associated with a pair $({n}_{{{{{{\rm{s}}}}}}},\tilde{D},\gamma ,\beta )$ of the seed graph, we can choose entries that are bigger than a threshold to get quasi-optimal parameters for the new graph. Specifically, based on the obtained entries, the parameters in the pair corresponding to each entry will be identified and then transferred to the new graph. In this sense, we can use an interval $[{\tilde{D}}_{{{{{{\rm{l}}}}}}},{\tilde{D}}_{{{{{{\rm{r}}}}}}}]$ to summarize the identified pairs and denote the mapping as ${D}^{{\prime} }\mapsto [{\tilde{D}}_{{{{{{\rm{l}}}}}}},{\tilde{D}}_{{{{{{\rm{r}}}}}}}]$, as shown in Fig. 2. To improve the result’s accuracy, the layer number *p* can be accordingly increased, with parameters obtained by above transfer strategy.

The presented quasi-optimal parameter database is expendable, as mentioned in the Step 5 of the data-driven QAOA. In the Substep 3, we obtain the identified quasi-optimal parameters. For one thing, these parameters can be directly applied to the QAOA calculation of the new graph. The new result can then be added to the current mapping table ${M}_{p}({n}_{{{{{{\rm{s}}}}}}}\mapsto {n}_{{{{{{\rm{t}}}}}}}^{{\prime} })$ as a new entry, which is associated with the pair $({n}_{{{{{{\rm{s}}}}}}},\tilde{D},\gamma ,\beta )$. For another, these parameters can also be decent initial guesses for further optimizing the parameters. Then based on the optimized result, a new pair $({n}_{{{{{{\rm{t}}}}}}}^{{\prime} },{D}^{{\prime} },{\gamma }^{{\prime} },{\beta }^{{\prime} })$ can be added to the database to provide potential parameters for new graphs, which is equivalent to the Substep 1. For the proposed parameter transfer strategy, we also provide the the pseudocode Algorithm 2 in Supplementary Methods.

In summary, the key idea of data-driven QAOA includes the following five steps referring to Fig. 2: First, by modeling the cyber-physical system into two normalized weighted graphs, we compute the normalized graph density from adjacency matrix *W*_adj_. Second, in the parameter transfer module, we first determine the seed graph size *n*_s_ and layer number *p*. Then, according to the mapping table *M*_*p*_(*n*_s_ ↦ *n*_t_) in Supplementary Fig. 1, we can obtain the quasi-optimal parameters (*γ*, *β*) from seed graphs, whose normalized densities can be expressed by an interval $[{\tilde{D}}_{l},{\tilde{D}}_{r}]$. Third, the transferred parameters (*γ*, *β*) are directly passed to the quantum circuit with multiple layers for QAOA. By measurement, it generates the probability distribution ${\left\vert {\alpha }_{k}\right\vert }^{2}$ in (5), from which we can obtain $C(\left\vert Z\right\rangle )$ and select a high approximation-ratio solution. Fourth, if a better performance is desired, we will further optimize (*γ*, *β*) for $C{\left(\left\vert Z\right\rangle \right)}_{\max }$. This step is optional. Fifth, the obtained pair (*n*, *D*, *γ*, *β*) can be used to develop an expandable quasi-optimal parameter database to provide quasi-optimal parameter for new target graphs. If fourth step is performed, by obtaining the optimized parameters, we can store a new pair (*n*_t_, *D*, *γ*_new_, *β*_new_) in a new mapping table for target graphs; otherwise, we can add one new entry $C(\left\vert Z\right\rangle )$ to the original mapping table, as exampled in Supplementary Fig. 1. This step is also optional.

### Numerical justification of the parameter transfer strategy

Since rigorously estimating the QAOA performace on generic weighted graphs is still an open question^42^, we provide numerical examples to justify the effectiveness of the parameter transfer strategy. We verify the efficacy of the transferred parameters from three aspects by comparing the approximation ratios with the ones obtained by QAOA using random parameters, QAOA using optimized parameters and GW algorithm.

For developing the mapping tables, we randomly generate 9 unweighted graphs with *n*_s1_ = 10 and 9 weighted graphs with *n*_s2_ = 24 as seed graphs, as given in Supplementary Table I. The 1710 non-planar target graphs with *n*_t_ = 24 are also randomly generated, including 714 unweighted graphs and 996 weighted ones. The justifications involve 39, 744 QAOA expectation value calculations and at least 16, 146, 548, 640 shots. We apply two classical optimizers based on the Newton and the Constrained Optimization BY Linear Approximation (COBYLA) methods^45^ to get the mean approximation-ratio of the seed graphs and their corresponding quasi-optimal parameters. The values for *p* = 1, 2, 3 are summarized in Supplementary Table I. These parameters (*γ*, *β*) provide the initial data for the expandable database as introduced in Fig. 2.

According to the Substep 2, we develop 12 mapping tables as examples, among which the 6 mapping tables in Supplementary Fig. 1 are for the unweighted seed graphs under both weighted and unweighted target graphs with *p* = 1, 2, 3, respectively; the other 6 mapping tables in Supplementary Fig. 2 are for the weighted seed graphs.

### Numerical justification—comparison with using random parameters

**Fig. 3:**
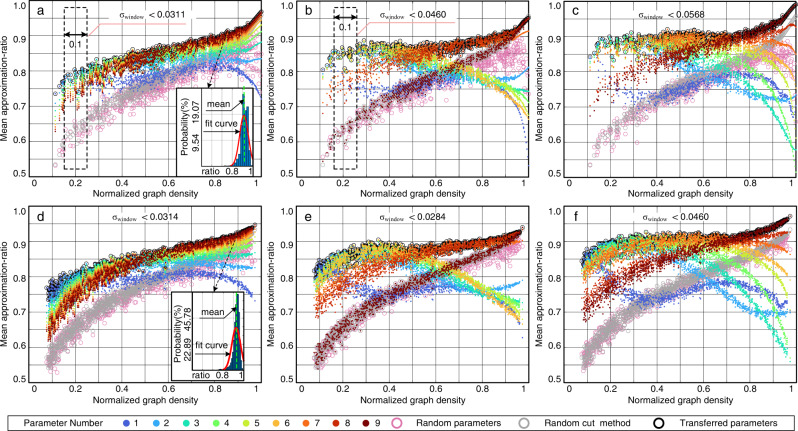
The mean approximation-ratio of randomly generated target graphs under parameters of seed graphs with *n*_s1_ = 10. **a** *p* = 1, unweighted target graphs. (inset) The detail approximation-ratio distribution of one data point. The solid red curve is the fitting normal distribution for the original distribution. The dotted green line is the mean value of the original distribution. **b** *p* = 2, unweighted target graphs. **c** *p* = 3, unweighted target graphs. **d** *p* = 1, weighted target graphs. (inset) The detail approximation-ratio distribution of one data point. **e** *p* = 2, weighted target graphs. **f** *p* = 3, weighted target graphs. The mean approximation-ratios are computed by the probability distribution, with details given in (5) and the Methods section “Measurement Outcomes for the Cost Function Value''. The probability with respect to approximation-ratio is fitted by normal distribution. The *σ*_window_ is the standard deviation of the scatters with the same color in the 0.1 scan window regarding to *D*.

**Fig. 4:**
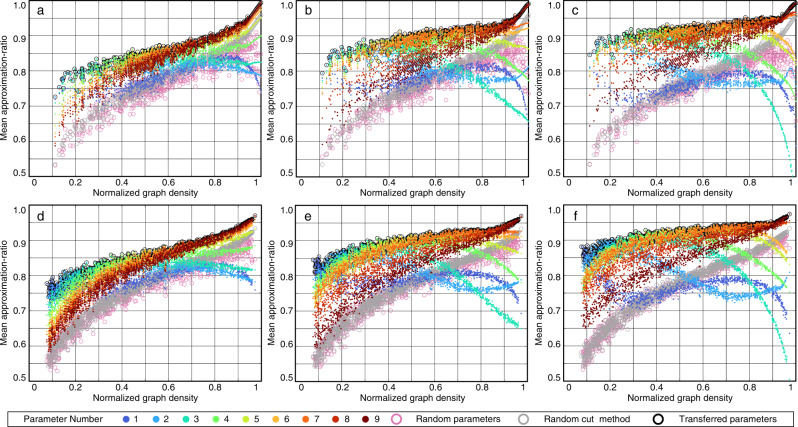
The mean approximation-ratio of randomly generated target graphs under parameters of seed graphs with *n*_s2_ = 24. **a** *p* = 1, unweighted target graphs. **b** *p* = 2, unweighted target graphs. **c** *p* = 3, unweighted target graphs. **d** *p* = 1, weighted target graphs. **e** *p* = 2, weighted target graphs. **f** *p* = 3, weighted target graphs.

We compare the QAOA results from the transferred parameters and from random parameters to verify the parameter transfer strategy. We apply each seed graph’s quasi-optimal parameters to the QAOA calculation for the 1,710 target graphs, respectively. Figure 3 summarizes the mean approximation-ratios of QAOA in the unweighted and weighted graphs for *p* = 1, 2, 3. In Fig. 3, each black circle represents the mean approximation-ratio for a target graph with the identified parameters obtained from the 9 groups parameters in the mapping tables developed from unweighted *n*_s1_ = 10 seed graphs. These black circles show the high approximation-ratios, which are 0.8501, 0.8911, 0.9125 in average for *p* = 1, 2, 3, respectively. Comparing these black circles with the pink circles showing the approximation ratios with random parameters, we can see that the parameter transfer strategy significantly improves the approximation ratio, especially for low density graphs, which verifies the effectiveness of the transfer strategy. In addition, in Fig. 4, we also observe high approximation-ratio with parameters transferred from weighted *n*_s2_ = 24 seed graphs.

Meanwhile, we have two insights in both Figs. 3 and 4. First, the significant improvement in low density graph is particularly appealing to power systems, which usually have low densities. Second, it is challenging for the random parameters method to efficiently handle barren plateaus, while our method can address this issue by providing quasi-optimal initial guesses^46^. Further explanations for the effectiveness of the transfer strategy are shown in “Methods” section “Findings for Transfer Principles”.

### Numerical justification—comparison with using optimized parameters

**Fig. 5:**
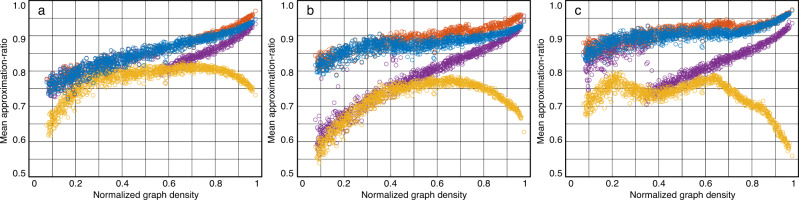
The mean approximation-ratio of quantum approximate optimization algorithm (QAOA) results after optimization. The blue scatters denote the approximation-ratio under the transferred parameters without optimization. The orange scatters denote the approximation-ratio under the transferred parameters with optimization. The yellow scatters denote the worst parameters in mapping table without optimization. The purple scatters denote the worst parameters in mapping table with optimization. **a** *p* = 1, weighted target graphs. **b** *p* = 2, weighted target graphs. **c** *p* = 3, weighted target graphs.

We verify that the transferred parameter can provide warm starting for QAOA. Figure 5 compares the mean approximation-ratio of QAOA using the transferred and unfavorable parameters in the mapping table with and without further optimization.

On the one hand, the transferred parameters can be decent initial guesses. Figure 5 shows that after further optimizing the transferred parameters (blue scatters), the result (orange scatters) has no significant improvement. On the other hand, those transferred parameters also can be quasi-optimal parameters. Most of blue scatters without optimization are better than the purple scatters, which are optimized from yellow scatters with lots of computational effort. There is a significant increment when comparing the transferred parameters with the worst parameters in the mapping table, as shown by the blue and yellow scatters in Fig. 5. These comparisons validate the effectiveness of the parameter transfer strategy.

In addition, the optimized parameters associated to the orange scatters in Fig. 5 can be adopted to expand the database.

### Numerical justification—comparison with the GW algorithm

**Fig. 6:**
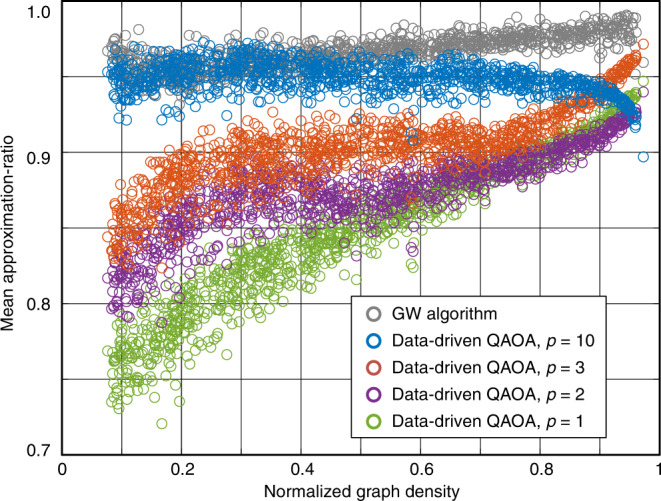
The comparison of the Goemans–Williamson (GW) algorithm and the data-driven Quantum Approximate Optimization Algorithm (QAOA) with different layer numbers without parameter optimization. The gray scatters denote the approximation-ratio of the GW algorithm. The blue, orange, purple, and green scatters denote the approximation-ratio of the QAOA algorithm without parameter optimization with layer number $p=10$, $p=3$, $p=2$, and $p=1$, respectively.

For further verifying our strategy can provide the promising results, we compare the results of using the GW algorithm with the ones via the data-driven QAOA. *p* = 1, 2, 3, 10 are adopted as examples. In Fig. 6, when *p* increases, the overall performance of transferred parameters increases. when *p* = 10, the transferred parameters without any optimization have better mean approximation-ratio than GW algorithm in the 113 graphs out of total 996 graphs. It is encouraging that, without any parameter optimization, the data-driven QAOA is competitive with GW algorithm. We expect that proper optimization and larger *p* could improve the approximation ratio further.

The drop trend of approximation ratio with *p* = 10 in the large graph density regime, as shown in Fig. 6, is due to the overfitting on the seed graphs with *D* = 0.9111 and *D* = 1. The 20 parameters in the 10-layer QAOA circuits could be excessive to be justified for some 10-vertex seed graphs. Notably, $p={{{{{\mathcal{O}}}}}}(\log (n))$ could be sufficient to obtain high approximation-ratio solutions^42^. The overfitting issue could be resolved in large seed graphs with *n* vertexes and $p={{{{{\mathcal{O}}}}}}(\log (n))$.

### Numerical examples of data-driven QAOA on power systems

We test and verify the data-driven QAOA on a typical power system. The physical system is a modified IEEE 24-bus system^47^, as given in Supplementary Fig. 3. Eleven DERs are integrated into the system. Considering the output fluctuations of DERs, the normalized graph density will correspondingly change over time. So, two operational scenarios with normalized graphs densities *D* = 0.0525 and *D* = 0.1053 are given as examples for the test. The communication network also has 24 vertices. Considering the dynamic data traffic in the network, two scenarios are considered as examples with *D* = 0.1143 and *D* = 0.3280, respectively. We provide the results from the following two aspects.

### Numerical example—test without the depolarizing noise

We carry out the test according to the steps given in Fig. 2. Based on the power flow calculation of the physical system or the data traffic measurement of the cyber layer, four normalized weighted graphs can be obtained. With the normalized graph densities, quasi-optimal parameters can be identified through the mapping table in Supplementary Fig. 1 for the QAOA calculation. To provide a comparison, Table 1 summarizes the mean approximation-ratios with all the parameters in the mapping table when *p* = 1, 2, 3 for the four graphs. The highlighted results emphasize that the best results based on the mapping table can be obtained with the transferred parameters. Thus, it justifies the effectiveness of the parameter transfer strategy.

**Table 1:** The QAOA results in four test graphs with different normalized graph densities

No.	seed graph $\tilde{D}$	$C(\left\vert Z\right\rangle )(D=0.0525)$			$C(\left\vert Z\right\rangle )(D=0.1053)$			$C(\left\vert Z\right\rangle )(D=0.1143)$			$C(\left\vert Z\right\rangle )(D=0.3280)$		
		*p* = 1	*p* = 2	*p* = 3	*p* = 1	*p* = 2	*p* = 3	*p* = 1	*p* = 2	*p* = 3	*p* = 1	*p* = 2	*p* = 3
1	0.2667	**0.6865**	**0.7393**	**0.7724**	**0.7840**	**0.8180**	**0.8315**	**0.7828**	**0.8260**	**0.8495**	0.8159	0.7581	0.7475
2	0.5333	0.6316	0.7059	0.7445	0.7577	0.7985	0.8216	0.7590	0.8150	0.8448	0.8448	0.8878	0.9107
3	0.6444	0.6135	0.6987	0.7368	0.7459	0.7941	0.8163	0.7431	0.8098	0.8384	**0.8466**	0.8940	0.9177
4	0.7333	0.6010	0.6991	0.7396	0.7373	0.7958	0.8176	0.7308	0.8110	0.8378	0.8450	0.8958	**0.9189**
5	0.8000	0.5920	0.7028	0.7460	0.7309	0.7965	0.8206	0.7216	0.8115	0.8363	0.8424	0.8963	0.9138
6	0.8667	0.5848	0.6892	0.7427	0.7258	0.7882	0.8209	0.7138	0.8027	0.8376	0.8393	**0.8965**	0.9146
7	0.9111	0.5793	0.6596	0.7259	0.7218	0.7769	0.8133	0.7077	0.7735	0.8306	0.8365	0.8669	0.9115
8	0.9556	0.5749	0.6448	0.6708	0.7183	0.7676	0.7876	0.7024	0.7537	0.7720	0.8337	0.8516	0.8607
9	1.0000	0.5710	0.4736	0.6386	0.7155	0.6593	0.7648	0.6979	0.6244	0.7454	0.8310	0.7617	0.8482

The bold expectation is the best value in the column.

**Fig. 7:**
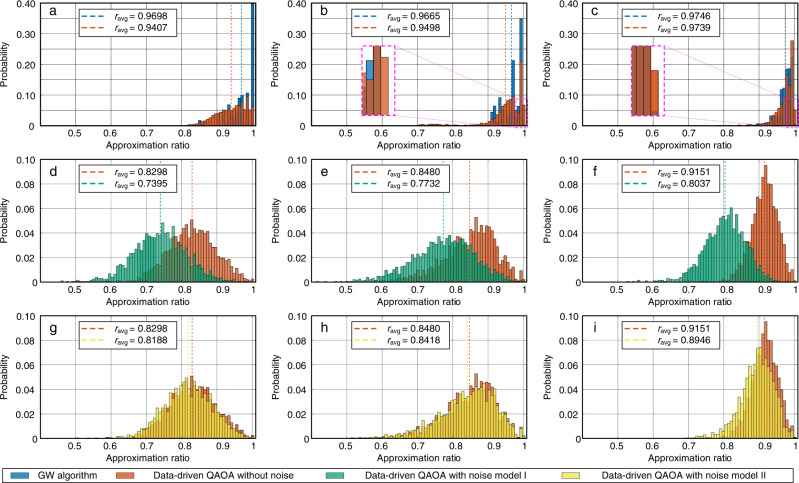
The comparison of the approximation-ratio distributions between the Goemans-Williamson (GW) algorithm and the date-driven Quantum Approximate Optimization Algorithm (QAOA) with different layer numbers. Noise model I is 0.1% depolarizing error on single-qubit gates and 1% depolarizing error on two-qubits gates; Noise model II is 0.01% depolarizing error on single-qubit gates and 0.1% depolarizing error on two-qubits gates. **a** *D* = 0.1053, *p* = 10, without noise. **b** *D* = 0.1143, *p* = 10, without noise. **c** *D* = 0.3280, *p* = 10, without noise. **d** *D* = 0.1053, *p* = 3, noise model I. **e** *D* = 0.1143, *p* = 3, noise model I. **f** *D* = 0.3280, *p* = 3, noise model I. **g** *D* = 0.1053, *p* = 3, noise model II. **h** *D* = 0.1143, *p* = 3, noise model II. **i** *D* = 0.3280, *p* = 3, noise model II.

We also compare the data-driven QAOA’s results with the GW algorithm. Figure 7a–c shows the approximation-ratio distributions of different normalized graph densities when *p* = 10, with the following findings. First, the results show that the approximation means are very close to those of the GW algorithm. More importantly, Fig. 7b, c show that the data-driven QAOA’s results are better than the GW algorithm, as there is at least ten times higher probability for the data-driven QAOA method than the GW algorithm to get the highest approximation ratio, as shown in the zoom-in details. In practice, we usually use the highest cut value as an approximate solution instead of the mean approximation-ratio. The data-driven QAOA can be better than the GW algorithm. Note these parameters are transferred from the mapping tables without any further optimization. Hence, according to the “Numerical Justification—Comparison with Using Optimized Parameters” section, when these parameters are used as initial guesses for further optimizing them, the better mean approximation-ratio are 0.9569, 0.9499, 0.9751 for the cases in Fig. 7a–c, respectively. Second, Table 1 shows that the mean approximation-ratio will increase as *p* increases; and thus, a relatively larger *p* is recommended for practical applications.

### Numerical example—test with the depolarizing noise

To verify the practicability of data-driven QAOA, we introduce the depolarizing noise on quantum gates to simulate the realistic noise on quantum simulators^48^. Two noise models are considered. The noise model I is with 0.1% depolarizing error on single-qubit gates and 1% depolarizing error on two-qubits gates, which is presently achievable. The noise model II is with 0.01% depolarizing error on single-qubit gates and 0.1% depolarizing error on two-qubits gates, which is achievable in the near term.

We carry out the numerical noise experiments on the test graphs. Figure 7d–i shows the examples of three graphs with *D* = 0.1053, *D* = 0.1143, and *D* = 0.3280, under the transferred parameters when *p* = 3. By comparing the approximation-ratio distributions and means between the results with and without noise, we can see that the mean approximation-ratios drop, with noise model I, as given in Fig. 7d–f. While with the smaller noise, the reduction of mean approximate-ratio is negligible, as shown in Fig. 7g–i. Therefore, it is feasible to run the data-driven QAOA on a NISQ quantum processor and address the Max-Cut problem in the practical power system in the near term.

## Conclusions

We present a data-driven QAOA to efficiently search for the maximum power or data sections in DER dominant power systems by leveraging quantum advantage. The parameter transfer strategy is designed to provide quasi-optimal parameters from seed graphs to target graphs. It addresses the challenge of obtaining the critical parameters in QAOA; and thus, highly improving the efficacy and efficiency of QAOA. In the transfer strategy, normalized graph density is utilized to bridge the seed and target graphs for developing an extendable mapping table. We have verified the transfer strategy by comparing our approximation ratios with those obtained by QAOA using random parameters, QAOA using optimized parameters and GW algorithm. The improvements show the superiority of proposed strategy, and encourage that the data-driven QAOA is competitive with GW algorithm. The parameter transferability has also been verified from two perspectives, namely between unweighted and weighted graphs and between small scale and large scale graphs as well as graphs with the same size. We simulate the presented method in a modified IEEE 24-bus system and demonstrated its effectiveness in finding the maximum sections with and without depolarizing noise. The presented method showcases the new computation of power systems when meeting quantum technology.

The potentials of this work include the following aspects. First, the application of the proposed data-driven QAOA with the parameter transfer strategy has a strong potential to be extended from Max-Cut problem to the general binary combinatorial optimization problems. Second, from the power engineering angle, our work has many potential applications including the optimization for smooth and quick black start, the unit commitment in large-scale systems, designing and managing the communication network, and among others. Therefore, as a promising early candidate for achieving quantum advantage on NISQ systems, our method can also be extended to address challenging issues in other complex engineered systems and eventually evolve into a formal quantum methodology.

## Methods

### Adiabatic approximation with QAOA

According to the adiabatic evolution theorem^31^, during the time interval [0, *T*], we can slowly change the system’s Hamiltonian from *H*_B_ to *H*_C_ and obtain the maximum energy state of *H*_C_ with high probability^29^. The changing process is exampled in below equation:

8 $$H\left(t\right)=\left[1-s\left(t\right)\right]{H}_{{{{{{\rm{B}}}}}}}+s\left(t\right){H}_{{{{{{\rm{C}}}}}}},$$

where $s\left(t\right)$ is a smooth function, $s\left(0\right)=0$ and $s\left(T\right)=1$. We then use Trotterization technique to emulate the evolution process^49^.

We discretize the total time interval [0, *T*] into intervals [*j*Δ*t*, (*j* + 1)Δ*t*] with small enough Δ*t*. Over the *j*^th^ interval, the Hamiltonian is approximately constant, i.e., *H*(*t*) = *H*((*j* + 1)Δ*t*). Therefore, the total time evolution operator *U*(*T*, 0) can be approximately discretized into 2*p* implementable operators with constant Hamiltonian^49^, as written in (9). The approximation will improve as Δ*t* gets smaller or, equivalently, as *p* gets bigger.

9 $$\begin{array}{rcl}U\left(T,0\right)=U(T,T-\Delta t)U(T-\Delta t,T-2\Delta t)\cdots U(\Delta t,0)\\ =\mathop{\prod }\limits_{j=0}^{p-1}U((\,j+1)\Delta t,j\Delta t)\approx \mathop{\prod }\limits_{j=1}^{p}{e}^{-iH\left(j\Delta t\right)\Delta t},\end{array}$$

where *U*((*j* + 1)Δ*t*, *j*Δ*t*) represents the time evolution from *j*Δ*t* to (*j* + 1)Δ*t*. Inserting (8) to (9) and using ${e}^{i({A}_{1}+{A}_{2})x}={e}^{i{A}_{1}x}{e}^{i{A}_{2}x}+{{{{{\mathcal{O}}}}}}({x}^{2})$, the time evolution operator can be expressed as,

10 $$U\left(T,0\right) 	 \approx \mathop{\prod }\limits_{j=1}^{p}{e}^{-i\left[\left(1-s\left(j\Delta t\right)\right){H}_{{{{{{\rm{B}}}}}}}+s\left(j\Delta t\right){H}_{{{{{{\rm{C}}}}}}}\right]\Delta t}\\ 	 \approx \mathop{\prod }\limits_{j=1}^{p}{e}^{-i\left(1-s\left(j\Delta t\right)\right){H}_{{{{{{\rm{B}}}}}}}\Delta t}{e}^{-is\left(j\Delta t\right){H}_{{{{{{\rm{C}}}}}}}\Delta t}+O(\Delta {t}^{2})\\ 	 \approx \mathop{\prod }\limits_{j=1}^{p}{U}_{{{{{{\rm{B}}}}}}}^{\left(j\right)}{U}_{{{{{{\rm{C}}}}}}}^{\left(j\right)},$$

where ${U}_{{{{{{\rm{B}}}}}}}^{\left(j\right)}$ and ${U}_{{{{{{\rm{C}}}}}}}^{\left(j\right)}$ are the time evolution operators, evolving the system under the Hamiltonian *H*_B_ for the time period of *β*_*j*_ = (1 − *s*(*j*Δ*t*))Δ*t* and the Hamiltonian *H*_C_ for the time period of *γ*_*j*_ = *s*(*j*Δ*t*)Δ*t*, respectively, as defined in (11).

11 $$\left\{\begin{array}{l}{U}_{{{{{{\rm{C}}}}}}}^{\left(j\right)}={e}^{-is\left(j\Delta t\right){H}_{{{{{{\rm{C}}}}}}}\Delta t}={e}^{-i{\gamma }_{j}{H}_{{{{{{\rm{C}}}}}}}} \hfill\\ {U}_{{{{{{\rm{B}}}}}}}^{\left(j\right)}={e}^{-i\left[1-s\left(j\Delta t\right)\right]{H}_{{{{{{\rm{B}}}}}}}\Delta t}={e}^{-i{\beta }_{j}{H}_{{{{{{\rm{B}}}}}}}} \end{array}\right.$$

In the evolution, $\left\vert \varphi \right\rangle$ represents the quantum state $\left\vert Z\right\rangle$ in the “QAOA for Max-cut Problem” section. Through applying ${U}_{{{{{{\rm{B}}}}}}}^{\left(j\right)}$ and ${U}_{{{{{{\rm{C}}}}}}}^{\left(j\right)}$ to the initial state $\left\vert \varphi (0)\right\rangle ={\left\vert +\right\rangle }^{\otimes n}$ alternately, we can compute the final state $\left\vert \varphi (T)\right\rangle$ in (12), which is expected to lead a high $C(\left\vert \varphi (T)\right\rangle )$ and collapse to maximum energy state after measurement.

12 $$\left\vert \varphi \left(T,\gamma ,\beta \right)\right\rangle =\mathop{\prod }\limits_{k=1}^{p}{U}_{{{{{{\rm{B}}}}}}}^{\left(j\right)}{U}_{{{{{{\rm{C}}}}}}}^{\left(j\right)}\left\vert \varphi \left(0\right)\right\rangle ,$$

where $\gamma =({\gamma }_{1},{\gamma }_{2},\ldots ,{\gamma }_{p})$ and $\beta =({\beta }_{1},{\beta }_{2},\ldots ,{\beta }_{p})$ need to be optimized, which requires expensive computational effort. For the original QAOA for Max-Cut problem, we also provide the the pseudocode Algorithm 3 in Supplementary Methods.

### Measurement outcomes for the cost function value

Quantum computers perform calculations based on the probability distribution of quantum states. In QAOA, we obtain the cost function value in (5) by sampling the quantum states, where ${\vert {\alpha }_{k}\vert }^{2}$ is the probability that the final state $\vert \varphi \rangle$ collapses on the computational basis $\vert {Z}_{k}\rangle$, as explained below.

First, we construct the quantum circuit of QAOA for the target graphs. In our study, the circuit is built in the Qiskit simulator^50^. The quantum circuit prepares the initial maximum energy state and computes the final state in (12) by using the quantum operators ${U}_{{{{{{\rm{B}}}}}}}^{\left(j\right)}$ and ${U}_{{{{{{\rm{C}}}}}}}^{\left(j\right)}$, whose implementations are illustrated in Fig. 2 and also shown in (13) and (14), respectively.

13 $${e}^{-i{\beta }_{k}{H}_{{{{{{\rm{B}}}}}}}}={e}^{-i{\beta }_{k}\mathop{\sum }\nolimits_{j = 1}^{n}{\sigma }_{j}^{x}}=\mathop{\prod }\limits_{j=1}^{n}{e}^{-i{\beta }_{k}{\sigma }_{j}^{x}}=\mathop{\prod }\limits_{j=1}^{n}{R}_{{{{{{\rm{X}}}}}}}^{(j)}(2{\beta }_{k}),$$

14 $${e}^{-i{\gamma }_{k}{H}_{{{{{{\rm{C}}}}}}}}={e}^{-i{\gamma }_{k}\sum {w}_{ij}\frac{I-{\sigma }_{i}^{z}{\sigma }_{j}^{z}}{2}}=\mathop{\prod}\limits_{\left\langle i,j\right\rangle \in E}{R}_{{{{{{\rm{ZZ}}}}}}}^{\left\langle i,j\right\rangle }(-{\gamma }_{k}{w}_{ij}),$$

where ${R}_{{{{{{\rm{X}}}}}}}^{(j)}$ means only applying *R*_X_ gate to the *j*^th^ qubit without changing other qubits; and ${R}_{{{{{{\rm{ZZ}}}}}}}^{\left\langle i,j\right\rangle }$ means only applying *R*_ZZ_ gate to the *i*^th^ and *j*^th^ qubits.

Second, we run the quantum circuit *N*_shot_ times and measure the final state for the probability distribution. Suppose the final state collapses on the $\vert {Z}_{k}\rangle$ with *N*_*k*_ times, the approximation of ${\vert {\alpha }_{k}\vert }^{2}$ is ${\vert {\tilde{\alpha }}_{k}\vert }^{2}={N}_{k}/{N}_{{{{{{\rm{shot}}}}}}}$. The approximation will improve as *N*_shot_ gets bigger. In this study, to get an accurate probability distribution and mean approximation-ratio, we perform *N*_shot_ = 2^19^ to approximate the distribution coefficients ${\vert {\alpha }_{k}\vert }^{2}$. In practice, 2, 048 shots works well and is recommended.

Third, we calculate the cost function $C(\vert \varphi \rangle )$ as shown in (5), which is the weighted summation of $C(\vert {Z}_{k}\rangle )$ with the non-zero coefficients ${\vert {\tilde{\alpha }}_{k}\vert }^{2}$. Since the standard deviation of $C(\vert \varphi \rangle )$ is in the order of $\sqrt{m}$^29^, *N*_shot_ is in the polynomial order. So, it is efficient to compute $C(\vert {Z}_{k}\rangle )$ with non-zero coefficients ${\left\vert {\tilde{\alpha }}_{k}\right\vert }^{2}$. Based on the calculation of $C(\vert {Z}_{k}\rangle )$ with non-zero coefficients ${\vert {\tilde{\alpha }}_{k}\vert }^{2}$, we select the $\vert {Z}_{{{{{{\rm{opt}}}}}}}\rangle$ with the maximal cut value as the final solution *Z*_opt_.

### Optimization of parameters

This subsection explains the parameter optimization involved in three perspectives of the “Numerical Justification of the Parameter Transfer Strategy” section and the “Numerical Examples of Data-Driven QAOA on Power Systems” section, where we need to optimize the parameters for high cost function values in seed graphs with *n*_s_ = 10 and in target graphs with *n*_t_ = 24, when *p* = 1, 2, 3, 10. The three perspectives are introduced below.

First, we get the optimal parameters for the seed graphs with *n*_s_ = 10 when *p* = 1, 2, 3 by classical optimization method. In our study, the Newton method is used to get the exact cost function values. Considering the non-convex landscapes of the cost function, we adopt multiple initial guesses for (*γ*, *β*) within [0, 2*π*]^*p*^ × [0, *π*]^*p*^. The number of initial guesses is designed in the polynomial order of *n* and *m*, which is proved to be sufficient for obtaining the optimal parameters^29^.

Second, we get the quasi-optimal parameters for the seed graphs with *n*_s_ = 10 when *p* = 10 by Fourier heuristic strategy^32^. In the Fourier strategy, the time complexity of obtaining quasi-parameters is reduced into ${{{{{\mathcal{O}}}}}}({{{{{\rm{poly}}}}}}(p))$ to avoid computational burden^32^, thus the parameters with high *p* can be obtained efficiently.

Third, after getting the transferred parameters, we further optimize them for verifying the efficacy of the transferred parameters. Specifically, we use the COBYLA method^45^ to further optimize the transferred parameters due to the fluctuations of the cost function values. As mentioned in Methods section “Measurement Outcomes for the Cost Function Value”, the quantum computer estimates the cost function values by sampling copies of output quantum state, which results in the fluctuations of the cost function values. The COBYLA method is used to address the optimization with this issue. The optimized results under the COBYLA method are given in Fig. 5 and Supplementary Table I. The COBYLA method is also used to carry out the optimization of transferred parameters for the test cases without the depolarizing noise.

Note that some certain gradient-based methods might also be able to find the quasi-optimal parameters with fluctuating cost function value, where the inaccurate gradient estimation may cause the escape from a local maximum and allow the converge towards a better one^41^.

### Test graphs preparation

Without losing generality, the test graphs are randomly generated, as introduced below. First, we randomly generate adjacency matrices. Second, we set the entries to be zero with different probability to ensure the densities of the test graphs spread over [0, 1]. Third, since there exist a polynomial algorithm for Max-Cut problem for the planar graph^51^, we check the generated graphs’ planar property by Kuratowski’s Theorem, such that all of the test graphs are not planar. Finally, we generate 11, 840 graphs, sort them by normalized graph densities, and then uniformly pick out 1710 graphs as test graphs.

### Findings for transfer principles

Here we show two important findings in Fig. 3 to further explain the transfer principles.

First, the mean approximation-ratio of QAOA for the target graphs are correlated to a Lipschitz continuous curve with respect to their normalized graph densities, although these target graphs are randomly generated. It is justified by a scan window with the size 0.1, as given in Fig. 3. The window shows that the upper limit of the standard deviations of the scatters with the same parameters is 0.057, which further indicates the scatters approximately follow a curve. It also indicates the normalized graph density is a effective metric. According to these curves, we can directly estimate the mean approximation-ratio of new graphs with parameters in the database. Thereafter the parameters with outstanding performance can be quickly identified for the QAOA circuit, avoiding the high computing effort.

Second, the parameters of seed graphs with low density perform better in target graphs with low density than in the ones with high density, and vice versa. This property is also uncovered in the mapping tables, where the yellow area denoting the high approximation-ratio will increase as *D* increases. Specifically, when the sizes of the seed and target graphs are very close, the yellow area will be around the diagonal line as shown in Supplementary Fig. 2; while, when the size of the target graph is much bigger than that of the seed graphs, the area will be above the diagonal line as shown in Supplementary Fig. 1. With this property, the quasi-optimal parameters can be effectively identified.

### Modeling the power system

We model the physical layer and then get multiple normalized weighted graphs through the power flow calculation when disturbances from DERs are considered. Power flow calculates the bus voltages for a given load, generation, and network condition, based on which the line powers (weights) can be obtained. The power flow equations are given in (15).

15 $${P}_{i}+j{Q}_{i}={\dot{V}}_{i}\mathop{\sum }\limits_{k=1}^{n}{\dot{y}}_{ik}^{* }{\dot{V}}_{k}^{* },$$

where * denotes conjugate, ${\dot{V}}_{i}\in {\mathbb{C}}$ is the *i*^th^ bus (vertex) voltage in the physical grid, ${P}_{i},{Q}_{i}\in {\mathbb{R}}$ is the injection active and reactive power of the *i*^th^ bus, and ${\dot{y}}_{ik}\in {\mathbb{C}}$ is the admittance of the line between the *i*^th^ bus and *k*^th^ bus.

After solving the power flow equations, we can obtain the complex power over each line. In our study, the apparent power is used as the edge weight. Due to the complex landscape of parameters^21^, the edge weight is then normalized. Thus, the modeling graph for the physical system is a normalized weighted graph.

The (15) describes the steady state of the physical power system. The DERs could be grid-forming or grid-following, and the corresponding bus types could be PV-bus, V*δ*-bus, or PQ-bus, respectively. The (15) does not involve the dynamic modeling of DERs but concludes them as the PQ-bus, PV-bus, or V*δ*-bus, depending on the microgrid system’s operational mode. When we consider the dynamics of power system, we can model the DERs in grid-following or grid-forming pattern. Then, differential algebra equations can be developed to describe the dynamics of the system. At each time step, the transient state describes the power delivery among buses. Then, our method can also be utilized to search the maximum power energy section. Thus, the current application on the steady state can then be extended to a general case.

The modeling graph of the cyber layer is based on the communication network data traffic that is flexible and random. In our study, we randomly generate *n*_t_ = 24 graphs to represent the communication network. In practical applications, we can monitor the data traffic to set up the edge weights for the cyber graphs.

### Depolarizing noise model

For demonstrating the potential of our method to be a promising candidate for achieving quantum advantage on NISQ systems, we introduce the depolarizing noise for the quantum gates in QAOA circuits. In our study, Qiskit^50^ is used to simulate the depolarizing noise and investigate the influences. The simulator needs to calculate the density matrix after each quantum gate to include the noise model, which costs exponentially more calculation resources than the noiseless vector simulation.

### The approximation-ratio distribution of GW algorithm

In Fig. 7a–c, we obtain the approximation-ratio distributions of the GW algorithm, as summarized below.

The GW algorithm relaxes the constraint of Max-Cut problem from discrete variables to the vectors on a unit sphere. The relaxed problem then becomes a semidefinite programming (SDP) problem. By solving the SDP problem, we obtain the optimal vector distribution. By randomly cutting the unit sphere into two parts, we correspondingly separate the vectors into two groups and obtain an approximate solution. When we cut the sphere several times, there is a guarantee that we have at least 0.878 expected approximation ratio.

Similarly to the shots in QAOA, with certain cut times, the GW algorithm outputs a probability distribution with respect to the approximation ratio, e.g., the approximation-ratio distribution. In the theoretical research, we usually compare the expectation of the approximation-ratio distribution to evaluate the algorithm performance, while in the practical applications to power systems, we can take the maximum approximation-ratio as the final approximation solution.

### Supplementary information

Supplementary Information

Description of Additional Supplementary Files

Supplementary Data 1

Supplementary Data 2

Supplementary Data 3

Supplementary Data 4

Supplementary Data 5

## Data Availability

The data that support the plots within this article and other findings of this study are available from the corresponding author upon reasonable request.

## Code availability

Code for this article is available from the corresponding author upon reasonable request.
