# Supplementary material for: Data-driven quantum approximate optimization algorithm for power systems
Source: Commun Eng. 2023 Mar 9;2:12. doi: 10.1038/s44172-023-00061-8 (PMC10955914; doi:10.1038/s44172-023-00061-8)
Supplement: Supplementary file 1 — Supplementary Information [file 44172_2023_61_MOESM1_ESM.pdf]

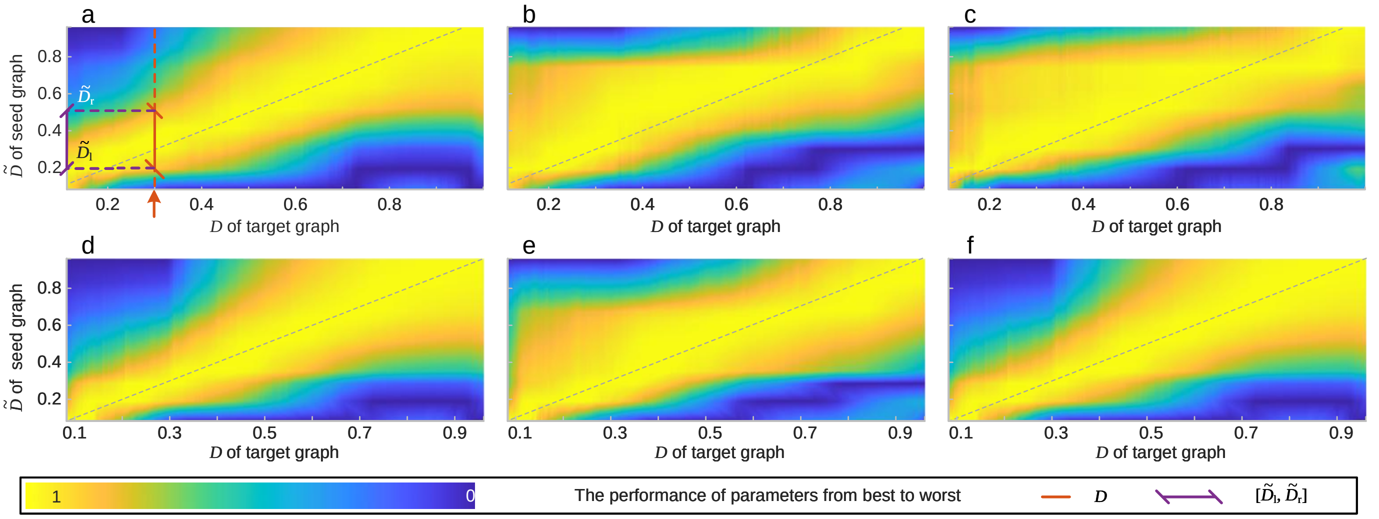

Supplementary Fig. 1. The mapping table developed by the parameters from unweighted seed graphs with $n_{\rm s1} = 10$. Each sub-figure is a mapping table for $D \mapsto [\tilde{D}_{\rm l}, \tilde{D}_{\rm r}]$ in the parameter transfer module of the proposed data-driven QAOA in Fig. 2. This mapping $D \mapsto [\tilde{D}_{\rm l}, \tilde{D}_{\rm r}]$ is performed based on the entries in the mapping table. The entries are the scaling approximation ratios, which can be obtained by applying the parameters from seed graphs to the QAOA for the target graphs, as shown in Fig. 3. **a** $p = 1$, unweighted target graphs. **b** $p = 2$, unweighted target graphs. **c** $p = 3$, unweighted target graphs. **d** $p = 1$, weighted target graphs. **e** $p = 2$, weighted target graphs. **f** $p = 3$, weighted target graphs. The procedure of getting $D \mapsto [\tilde{D}_{\rm l}, \tilde{D}_{\rm r}]$ is that we first fix the horizontal axis as shown in the orange arrow according to D and then find the interval $[\tilde{D}_{\rm l}, \tilde{D}_{\rm r}]$ as shown in purple line according to the contour. After mapping, we identify the quasi-optimal parameters from the seed graphs whose $\tilde{D}$ are within $[\tilde{D}_{\rm l}, \tilde{D}_{\rm r}]$.

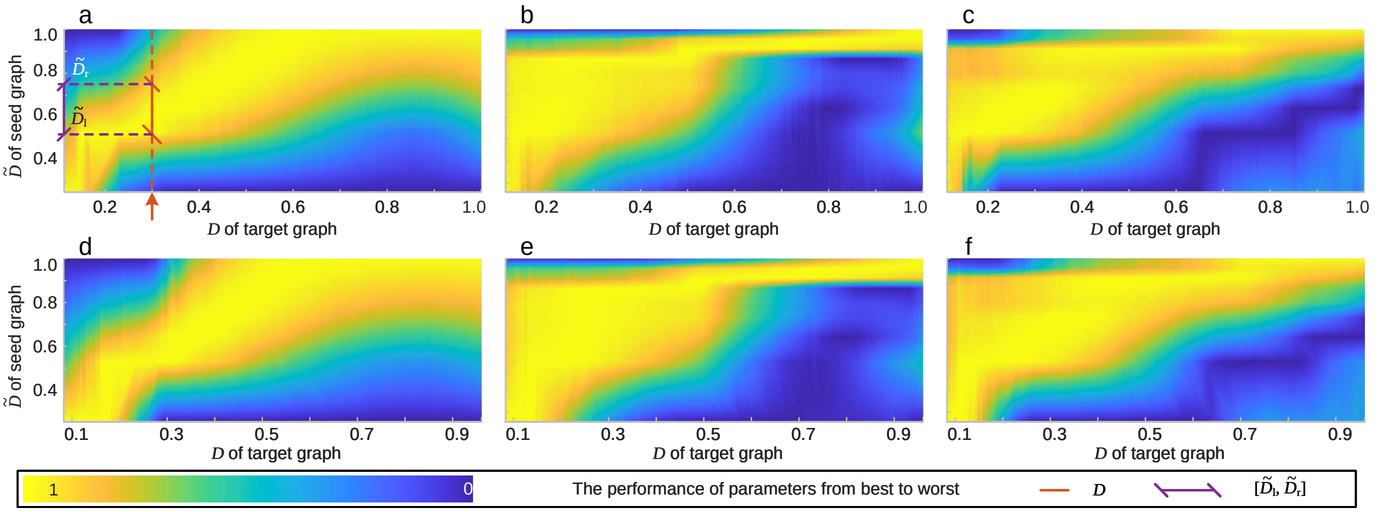

Supplementary Fig. 2. The mapping table developed by the parameters from weighted seed graphs with $n_{\rm s2} = 24$. Each sub-figure is a mapping table for $D \mapsto [\tilde{D}_{\rm l}, \tilde{D}_{\rm r}]$ in the parameter transfer module of the proposed data-driven QAOA in Fig. 2. **a** $p = 1$, unweighted target graphs. **b** $p = 2$, unweighted target graphs. **c** $p = 3$, unweighted target graphs. **d** $p = 1$, weighted target graphs. **e** $p = 2$, weighted target graphs. **f** $p = 3$, weighted target graphs. The developing procedure of these mapping tables is the same with that of the mapping tables in Supplementary Fig. 1. Note that since we identify the parameters from $n_{\rm s2} = 24$ seed graphs and apply them to the same size $n_{\rm t} = 24$ target graphs, the maximal performances are along the diagonal dash line.

Supplementary TABLE I
The Parameters Performance of Seed Graphs with Different Layer Numbers p and Vertex Size n_{s}

seed graph	$C(\|Z\rangle)$ when $n_s = 10$				$C(\|Z\rangle)$ when $n_s = 24$			
	D	$p=1$	$p=2$	$p=3$	D	$p=1$	$p=2$	$p=3$
1	0.2667	0.7783	0.8727	0.9287	0.0831	0.7395	0.8117	0.8573
2	0.5333	0.8537	0.9193	0.9516	0.1901	0.7931	0.8526	0.8866
3	0.6444	0.8447	0.9009	0.9296	0.2869	0.8122	0.8681	0.8950
4	0.7333	0.8182	0.8601	0.8885	0.3527	0.8363	0.8872	0.9114
5	0.8000	0.8797	0.9206	0.9493	0.5163	0.8518	0.8974	0.9178
6	0.8667	0.8672	0.8943	0.9170	0.6256	0.8793	0.9168	0.9325
7	0.9111	0.9044	0.9223	0.9396	0.6756	0.8743	0.9105	0.9259
8	0.9556	0.9420	0.9553	0.9639	0.8316	0.9152	0.9261	0.9353
9	1.0000	0.9804	0.9977	0.9999	0.9608	0.9588	0.9588	0.9661

We randomly generate several seed graphs, including 9 unweighted $n_{\mathrm{s}1} = 10$ graphs with D spreading over $[0.2667, 1]$, and 9 weighted $n_{\mathrm{s}2} = 24$ graphs with D spreading over $[0.0831, 0.9608]$. In each seed graph, we use the classical optimizers mentioned in Methods Section C to obtain the quasi-optimal parameters, which have better approximation ratio as the layer number p increases.

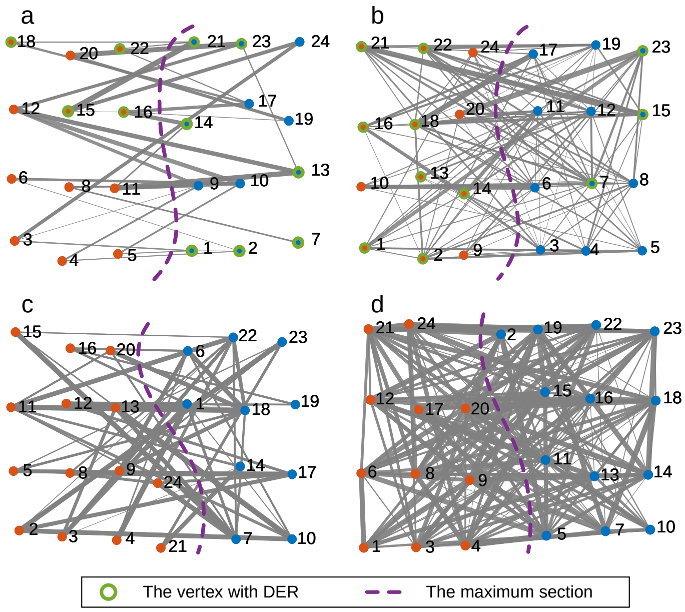

Supplementary Fig. 3. The four scenarios of the test power system. **a** $D = 0.0525$, physical layer. **b** $D = 0.1053$, physical layer. **c** $D = 0.1143$, cyber layer. **d** $D = 0.3280$, cyber layer.

Supplementary Methods

Algorithm 1: The data-driven QAOA

Input : The seed graph size $n_{\rm s}$; The target graph size $n_{\rm t}$; The normalized $n_{\rm t} \times n_{\rm t}$ adjacent matrix $W_{\rm adj}$ from Section II; The mapping table $M_p(n_{\rm s} \mapsto n_{\rm t})$ in the expandable quasi-optimal parameter database; The layer number p; The shot times $N_{\rm shot}$.

Output : The transfer parameters (γ, β) or the optimized parameters (γ', β'); The approximation solution $Z_{\rm opt}$ for the Max-Cut problem in target graph.

```
(γ, β) ← paraTransfer(n_s, n_t, W_adj, p); /* The
  parameter transfer strategy in Algorithm 2 */
if Optimize the transfer parameters is needed then
    Initialize N_shot × 1 index array I;
    while the stop conditions of optimization do
        F ← 0; Initialize N_k ← 0;
        for j ← 1 to N_shot do
            I[j] ← QAOAShot(γ, β);
            N_I[j] ← N_I[j] + 1;
        for k ∈ I do
            |α̃_k|^2 ← N_k/N_shot;
            F ← F + |α̃_k|^2 C(Z_k);
        Iterate the parameter γ and β;
    D ← 2 ∑ w_ij/[n_t(n_t − 1)];
    Expand the database by adding the pair
     (n_t, D, γ, β) into mapping table M_p(n_t ↦ n) to
     provide more quasi-optimal parameters for the
     other potential target graph. This step is
     optional.
else
    Expand the database by updating one element
     with value F indexed by D column and D̃ row
     in the mapping table M_p(n_s ↦ n_t) to improve
     the accuracy of original mapping table. This
     step is optional.
Initialize N_k ← 0;
for j ← 1 to N_shot do
    I[j] ← QAOAShot(γ, β);
    N_I[j] ← N_I[j] + 1;
Obtain K ⊆ I for k ∈ K having high N_k;
C_best ← 0;
for k ∈ K do
    if C_best < C(Z_k) then
        C_best ← C(Z_k); Z_opt = Z_k;
```

Algorithm 2: The parameter transfer strategy

Function `paraTransfer`(n_s, n_t, W_{adj}, p)**:**
- **Data:** The seed graph size n_s; The target graph size n_t; The normalized $n_t \times n_t$ adjacent matrix W_{adj}; The mapping table $M_p(n_s \mapsto n_t)$ in the expandable quasi-optimal parameter database; (We assume the initial database is already established according to the Substep 1 in Section V.)
- **Result:** The transferred parameters (γ, β);
- $D' \leftarrow 2\sum w_{ij}/[n_t(n_t-1)]$;
- Search the sorted column indices of $M_p(n_s \mapsto n_t)$ for D, which is near D'.
- In the column indexed by D, the elements with row indices $\tilde{D} \in [\tilde{D}_l, \tilde{D}_r]$ have high value.
- For each element indexed by D in column and $\tilde{D} \in [\tilde{D}_l, \tilde{D}_r]$ in row, there is a corresponding pair $(n_s, \tilde{D}, \gamma, \beta)$, where the (γ, β) is the transfer parameters.

Algorithm 3: The original QAOA for Max-Cut

Input : The formulated normalized adjacent matrix W_{adj} in Section II; Layer number p; The shot times N_{shot}; The initial guesses number N_{ig}.

Output : The optimized parameters (γ, β). The approximation solution Z_{opt}.

Function `QAOAShot`(γ, β)**:**
- **Data:** $n \times n$ adjacent matrix W_{adj}; The quantum circuit parameters $\gamma = (\gamma_1, \gamma_2, \ldots, \gamma_p)$ and $\beta = (\beta_1, \beta_2, \ldots, \beta_p)$.
- **Result:** An index k of the measured $|Z_k\rangle$.
- Initialize the n qubits quantum state as $|0\rangle^{\otimes n}$;
- **for** $i \leftarrow 1$ **to** n **do**
  - Apply Hadamard gate to the ith qubit;
- **for** $q \leftarrow 1$ **to** p **do**
  - **for** $i \leftarrow 1$ **to** n **do**
    - **for** $j \leftarrow 1$ **to** n **do**
      - **if** $w_{ij} \neq 0$ **then**
        - Apply $R_{ZZ}(-w_{ij}\gamma_q)$ to the ith and j^{th} qubit;
  - **for** $i \leftarrow 1$ **to** n **do**
    - Apply $R_X(2\beta_q)$ to the ith qubit;
- Measure the quantum state on basis;
- Output the index k of the measured $|Z_k\rangle$;

for $i \leftarrow 1$ **to** N_{ig} **do**
- Initialize the parameters γ and β;
- Initialize $N_{shot} \times 1$ index array I;
- $F_{best} \leftarrow 0$; **while** *the stop conditions of optimization* **do**
  - Initialize $N_k \leftarrow 0$;
  - **for** $j \leftarrow 1$ **to** N_{shot} **do**
    - $I[j] \leftarrow$ `QAOAShot`(γ, β);
    - $N_{I[j]} \leftarrow N_{I[j]} + 1$;
  - $F[i] \leftarrow 0$;
  - **for** $k \in I$ **do**
    - $|\tilde{\alpha}_k|^2 \leftarrow N_k/N_{shot}$;
    - $F[i] \leftarrow F[i] + |\tilde{\alpha}_k|^2 C(Z_k)$;
  - Iterate the parameter γ and β;
- **if** $F_{best} < F[i]$ **then**
  - $F_{best} \leftarrow F[i]$; $\gamma_{best} \leftarrow \gamma$; $\beta_{best} \leftarrow \beta$;

Initialize $N_k \leftarrow 0$;

for $j \leftarrow 1$ **to** N_{shot} **do**
- $I[j] \leftarrow$ `QAOAShot`$(\gamma_{best}, \beta_{best})$;
- $N_{I[j]} \leftarrow N_{I[j]} + 1$;

Obtain $K \subseteq I$ for $k \in K$ having high N_k;

$C_{best} \leftarrow 0$;

for $k \in K$ **do**
- **if** $C_{best} < C(Z_k)$ **then**
  - $C_{best} \leftarrow C(Z_k)$;$Z_{opt} = Z_k$;
