# Supplementary material for: Data-driven quantum approximate optimization algorithm for power systems
Source: Commun Eng. 2023 Mar 9;2:12. doi: 10.1038/s44172-023-00061-8 (PMC10955914; doi:10.1038/s44172-023-00061-8)
Supplement: Supplementary file 2 — Description of Additional Supplementary Files [file 44172_2023_61_MOESM2_ESM.pdf]

Description of Additional Supplementary Files

File name: Supplementary Data 1

Description: The data for Fig. 3

File name: Supplementary Data 2

Description: The data for Fig. 4.

File name: Supplementary Data 3

Description: The data for Fig. 5.

File name: Supplementary Data 4

Description: The data for Fig. 6.

File name: Supplementary Data 5

Description: The data for Fig. 7.
